# Anti-inflammatory Effect of Metronidazole in Hospitalized Patients with Pneumonia due to COVID-19

**DOI:** 10.22037/ijpr.2021.114567.14917

**Published:** 2021

**Authors:** Muhanna Kazempour, Hossein Izadi, Arezoo Chouhdari, Morteza Rezaeifard

**Affiliations:** a *Department of Rheumatology, Loghman Hakim Hospital, Shahid Beheshti University of Medical Sciences, Tehran, Iran. *; b *Department of Internal Medicine, Shohada Gomnam Hospital, Shahid Beheshti University of Medical Sciences, Tehran, Iran. *; c *Skull Base Research Center, Loghman Hakim Medical Center, Shahid Beheshti University of Medical Sciences, Tehran, Iran. *; d *Biochemistry Institute, Faculty of Medical Sciences, Tarbiat Modares University, Tehran, Iran.*

**Keywords:** Coronavirus disease, COVID-19, Cytokines, Interleukin, Metronidazole

## Abstract

Metronidazole (MTZ) can decrease the levels of several cytokines. This research aimed at the investigation of the anti-inflammatory impact of MTZ in COVID-19. A randomized, single-blind clinical trial for comparing the anti-inflammatory effect of MTZ in two eligible groups of adult patients with lower respiratory tract involvement due to Covid-19 treated with a standard national method with or without MTZ was performed. Inflammatory markers were measured as the primary outcome in two groups. Oxygen saturation, length of hospital stays, and mortality of patients were evaluated as secondary outcomes. Among 44 patients with lower respiratory tract due to Covid-19, 20(45.5%) were randomly allocated in group A with the current standard treatment plus the MTZ tablet for 7 days orally and 24 (54.5%) in group B with the current standard treatment. The mean of *ESR* in group A was statistically significantly lower than that of group B on the seventh day (A: 38.25 ± 18.75 *vs.* B: 47.67 ± 26.41, *p* = 0.02). Moreover, the mean of *IL6* diminished significantly in both A (*p* = 0.01) and B (*p* = 0.01) groups on the seventh day compared to the first day. The decrease of *TNF* was not significant in any of the groups A (*p* = 0.3) and B (*p *= 0.4) from the 7^th^ day to the first day. No significant difference was not found between group A and group B groups on the *CRP* level (*p *= 0.1). Findings of this study showed the anti-inflammatory impact of MTZ in the patient with lower respiratory inflammation due to COVID-19.

## Introduction

In December 2019, researchers recognized a new coronavirus called severe acute respiratory syndrome coronavirus (SARS-CoV-2) as the cause of the respiratory disease that has been known as Covid-19(1). This virus results in infection involving the upper respiratory tract and causes severe pneumonia, respiratory distress, or even mortality (2). Therefore, researchers assessed several antiviral parameters to cure Covid-19; however, they failed to treat the patients (3-4). One reason may be the predominance of host inflammatory responses in patients with pulmonary hypoxia over viral pathogenicity (5). Viral infection may stimulate the overactivation of the immune system. Moreover, multi-organ involvement, coagulopathy, cytopenia, the increased level of ferritin, AST, as well as ALT have been reported in COVID–19. According to the results, there is a correlation between the greater inflammatory 

biomarkers and proinflammatory cytokines like interleukin 6, C–reactive protein (*CRP*), severe lymphopenia, ferritin as well as the higher lactate dehydrogenase (*LDH*) levels and the greater rates of ICU admission and mortality (6-8). Moreover, researchers found a considerable difference between severe and mild cases of COVID-19 in interleukin-6 (*IL-6*), thrombin time (*TT*), -Dimer, glucose (*GLU*), C-reactive protein (*CRP*), and fibrinogen (*FIB*) (9).In addition, in the hospitalized patients, Huang et al.’s analyses revealed greater initial level of plasma of a majority of proinflammatory cytokines like interleukin *(IL)1B, IL7, IL1RA, IL9, IL8, IL10*, granulocyte-colony stimulating factor (*GCSF*), basic fibroblast growth factor (*FGF*), interferon(*IFN*)**γ**, granulocyte-macrophage colony-stimulating factor (*GMCSF*), monocyte chemoattractant protein 1 (*MCP1*), *IFN-****γ*** inducible protein 10 (IP10), MIP1B, macrophage inflammatory protein (MIP)1A, tumor necrosis factor (*TNF*)-α, vascular endothelial growth factor (*VEGF*)as well as platelet-derived growth factor (*PDGF*). Furthermore, they observed the greater plasma level of *IL7, IL2, GCSF, IL10, MCP1, IP10, TNF-α *as well as MIP1A patients admitted to ICU (10). Hence, it is necessary for the clinicians to especially consider the impacts of immune-inflammatory factor release and select the better medication for curing the covid-19 infections. It is notable that 

MTZ has been proposed as one of the efficacious antimicrobial agents against diverse micro-organisms like anaerobic protozoa and bacteria. It also can be used to treat Crohn’s disease. The mechanism of action of MTZ has not been fully understood, but it appears that nitro group reduction by anaerobic organisms is the cause of its antimicrobial and cytotoxic effects (11). Seyed hamzeh et al. showed that *IL-12* increases in covid-19. MTZ can prevent *IL-12* from binding to the IL-12 receptors by modifying the surface area and volume of IL-12 (12). MTZ via its interaction with a nitroreductase homolog, acts as biocidal agents. With regard to the investigations, MTZ is capable of decreasing C-reactive protein (*CRP*) level, interleukin 8, *IL1B, IL6, IL12, IL1fi,* interferon (*IFN*)γ, tumor necrosis factor (*TNF)*-α. These parameters Increased during COVID-19 infection (13). 

Therefore, the present research intended to investigate the anti-inflammatory and therapeutic effects of metronidazole in COVID-19 infection.

## Experimental

*Research design and ethics statements*

 The present single-blind, randomized controlled clinical trial was conducted on adults hospitalized with Covid-19. Therefore, this survey has been recorded in the Iranian registry of clinical trials (N: IRCT202006080447686N1) and the ethics committee of Shahid Beheshti University of Medical Sciences, Tehran, Iran (ethics committee number: IR.SBMU.RETECH.REC.1399.157 verified it.

*Patients*

 The present clinical trial was performed at the Shohada Gomnam Hospital as a center for admission to Covid-19 patients in May 2020. Covid-19 infection was confirmed by clinical evaluation and lung CT scan regardless of RT-PCR result. Inclusion criteria were hospitalized patients≥18years with moderate COVID-19 pneumonia who signed an informed consent form to each part of treatment before the study and agreed not to participate in another survey before ending this research. Exclusion criteria were one history of an allergic reaction to MTZ, pregnancy, or positive pregnancy test so breastfeeding, a severe disease that needs ICU admission or intubation at the timing of admission, hemoperfusion, and patients who were discharged earlier than one week due to recovery. We allocated the patients randomly to the intervention and control groups while they did not know which group they belonged to20 patients in the intervention group received 250 mg MTZ tablets orally every 6 h for 7 days in addition to the standard medications based on national guidelines and clinical judgment of the treating physician. The national treatment protocols included hydroxychloroquine (200 mg twice daily), Lopinavir/Ritonavir (400/100 mg two times daily), and Ribavirin (1200 mg twice daily). Patients were not given glucocorticoids. Oxygen saturation was constantly checked through pulse oximetry, and patients received oxygen by nasal cannula, facial mask, and reserve mask if needed. As well as if noninvasive ventilation (NIV) or intubation was needed, patients were referred to ICU.

*Primary and secondary outcomes*

 We serially evaluated laboratory markers of inﬂammation as a primary outcome within 7 days from initiation of treatment. Blood samples were obtained to assess outcomes.*IL-6, TNF, CRP, ESR*, Ferritin, WBC, Platelet, AST, ALT, ALP, LDH, and D-Dimer were measured on the first and seventh days. Oxygen saturation without receiving oxygen is measured several times per day, and the lowest oxygen saturation is considered. Oxygen saturation**, **length of hospital stays, and mortality of patients were measured as secondary outcomes.

*Statistical Analyses*

This stage dealt with descriptive analyses with the mean, median for continuous, standard deviation (SD), frequency and percentage for qualitative data. Independent t-test, Chi-squared test, Fisher’s exact, and Mann–Whitney U test were employed to compare the basic variables between the two groups. Kendall Tau-b correlation coefficient was calculated to compare the baseline *CRP* between the two groups. To investigate the differences of the laboratory tests within each group during one week of hospitalization, we used a paired *t*-test. To investigate the difference between the two groups regarding *CRP* during one week, a marginal proportional odds model was applied using a generalized estimating equation (GEE) was applied considering a proportional odds model (the interaction was not significant in this model). To compare the quantitative variables between the intervention group and the control group on the seventh day of hospitalization by adjusting the baseline value, we used ANCOVA, and to examine changes in each group, as mentioned earlier, a paired t-test was used. The significance level was considered 0.05 for all statistical analyses and SPSS software version 20 was used for data analysis.

## Results

In this study, 44 patients with Covid 19 with a mean age of 63.05 ± 16.32 and range (18-93) years were randomly assigned into two treatment groups: Group A, 20 (45.5%), and group B, 24 (54.5%) patients. Eighteen (40.9%) and 26 (59.1%) patients were female and male, respectively. Any significant differences were not found between the mean age (*p* = 0.5) as well as sex (p=0.9) between the two groups of study. Seventy-five percent of the patients in group A and 70.8% of the cases in group B experienced the underlying disease. Moreover, any differences were not observed between the study groups concerning underlying disease (*p *= 0.7)) (Table 1).

The drugs for each group were prescribed are shown in Table 2. The proportion of patients receiving antiviral agents (*p* = 0.4) was statistically insignificant between the intervention and control groups. Two patients in each group were admitted to the ICU, not statistically significant (*p* = 0.9). The median length of hospital and ICU-stay of patients in groups A and B was 6 (3%), 2 (10%)days and 8 (6%), 2 (8.3%) days, respectively, which did not differ in each group (*p* = 0.9). None of the hospitalized patients in the two treatment groups expired.

**Figure 1:**
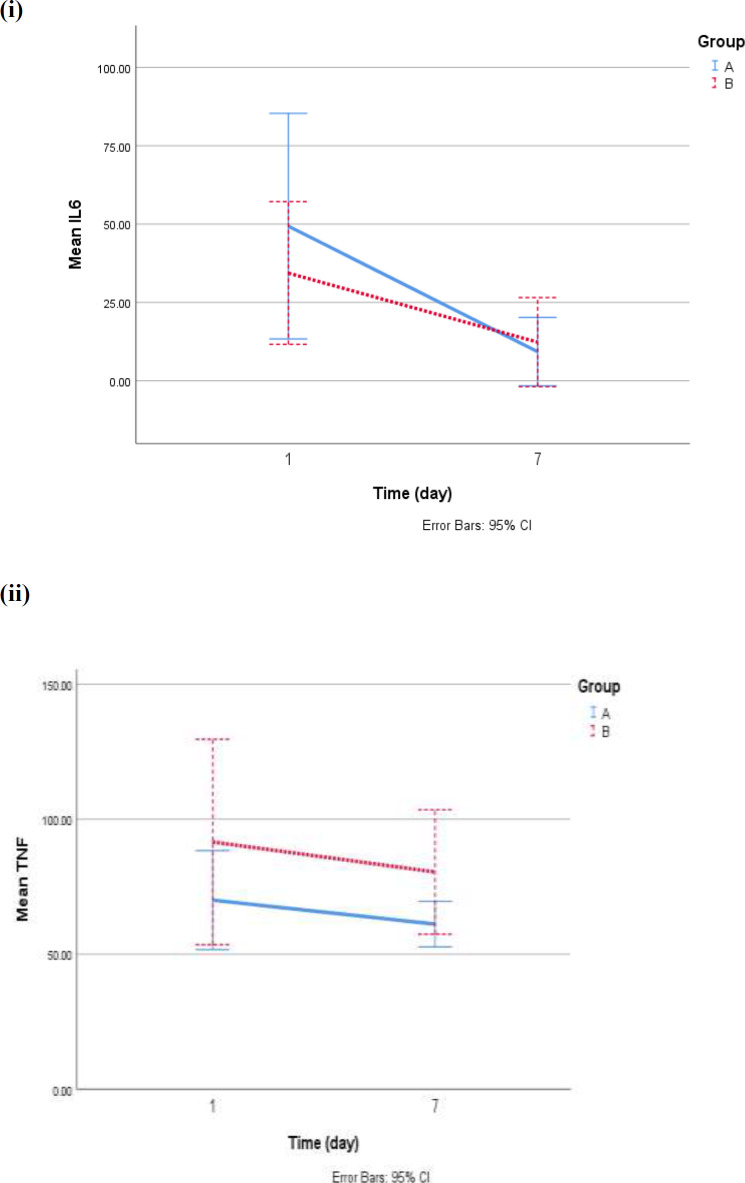
*IL6* (i) and *TNF* (b) changes according to study groups

On the first day of hospitalization, any significant differences were not observed between the control ad intervention group in the mean levels of interleukin 6(*IL6*) (*p* = 0.4) as well as *TNFα* (*p* = 0.3). The mean of *IL6* in groups A and B on the 7^th ^day were significantly lower than on the first day (mean decrement in the group: A: 40.06 ± 49.08, *p* = 0.01 and in group B: 22.04 ± 33.62, *p* = 0.01. The mean reduction of *TNF* on the seventh day compared to the first day was not significant in group A (*p *= 0.3) and group B (*p *= 0.4). Therefore, analysis of covariance (ANCOVA) with adjustment for *IL6* and *TNFα* on the first day, age, sex, antiviral agents showed no significant difference in the level of interleukin (*p *= 0.1) and *TNF* (*p *= 0.2) on the seventh day between two groups (Figure 1).

In addition, statistically, any significant differences were not observed in mean *ESR* (*p* = 0.385), ferritin (*p* = 0.524), and D. Dimer (*p *= 0.311) between the control and intervention group at the beginning of this investigation. On the seventh day*, ESR* in group B had a significant increase compared to the first day (*p* = 0.003), but *ESR* in group A decreased, even though there was no significant decrease (*p *= 0.836). The results of the analysis of covariance showed that the mean *ESR* in group B was significantly higher on the seventh day than group A (*p* = 0.021). The mean ferritin in both groups receiving A (*p* = 0.171) and B treatment (*p* = 0.188) on the seventh day from the first day was not statistically significant. Also, according to the ANCOVA result, statistically, no significant differences in mean ferritin between the intervention and control groups on the seventh day were seen (*p* = 0.351). As well as on the seventh day, the mean of D.Dimer in both study groups to the first day was not statistically different (*p *> 0.05 in both groups), and there was no significant difference in the mean of D.Dimer on the seventh day between the two groups based on ANCOVA result covariance (*p* = 0.898). (Table 3).

Based on the findings in Table 4, at the beginning of the survey, any significant differences were not observed in the mean of any of the variables between the control group and intervention group (*p* > 0.05). The mean oxygen saturation level on the seventh day compared to the first day in both group A (*p* = 0.009) and group B (*p* = 0.001) significantly increased, but the ANCOVA result did not statistically any significant differences in mean oxygen saturation on the seventh day between the two groups (*p* = 0.8). Baseline values of platelets, PDW, MPV, WBC count, lymphocyte count, ALT, AST, ALP, BIL, and LDH, did not statistically differ between the study groups. Furthermore, there was no statistically observed significant difference between the control and intervention groups based on ANCOVA results regarding platelets, PDW, MPV, WBC count, lymphocyte count, ALT, AST, ALP, BIL, and LDH values.

**Figure 2:**
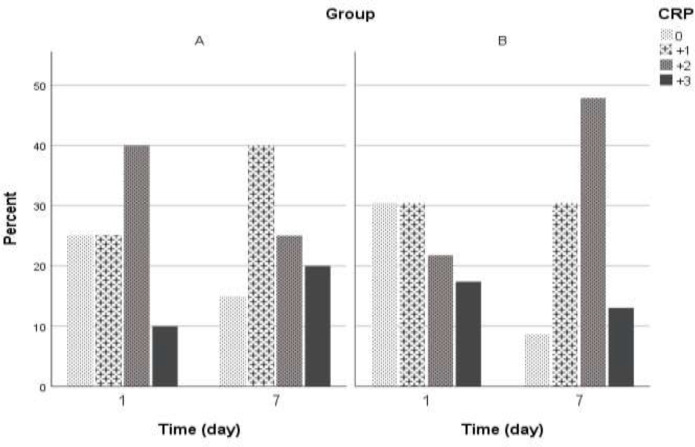
*CRP* changes during 1^st^ and 7^th^ hospitalized days according to the study group

**Figure 3:**
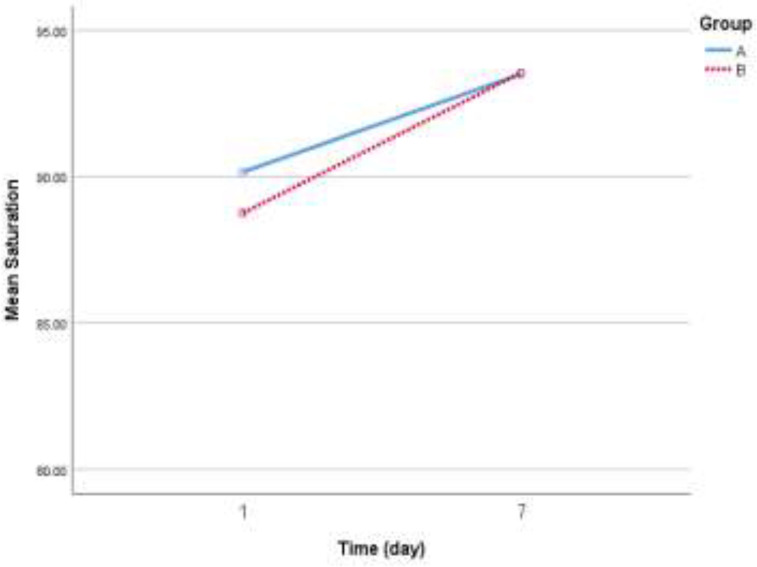
Changes in oxygen saturation during one week of hospitalization

On the first day of hospitalization in group A, 25% and in group B, 30.4% of patients had *CRP* = 0. We observed no statistically significant differences in *CRP* level between study groups (*p* = 0.7). The *CRP* changes within one week were shown in Figure 2. Statistically, any significant differences were not observed between the control group and intervention group with control of the effect of age, sex, anti-inflammatory, antiviral agents, and atorvastatin (OR = 0.663; 95%CI= 0.361 – 1.22; *p *= 0.1). The likelihood of higher *CRP* decreased during one week, but we found no significant decrement statistically (OR = 0.451; 95% CI:0.163 - 1.25; *p* = 0.4). Changes in oxygen saturation during one week of hospitalization were shown in Figure 3.

## Discussion

In this study, 20 patients with the standard treatment addition to the MTZ (group A) and 24 patients who received only standard treatment (group B) were compared to reduce inflammatory factors within 7 days. As previously stated in covid-19 patients, decreased oxygen saturation and laboratory abnormalities included high levels of *IL-6, TNF, ESR, CRP*, Ferritin, AST, ALT, and LDH was observed. We focused on the anti-inflammatory effect of MTZ in patients with covid-19 and found that *ESR* not only did not decrease in group B, but also the mean *ESR* in group B was significantly higher on the seventh day than group A (*p* = 0.021). It showed that MTZ inhibited *ESR* in patients with covid-19. *IL-6 and (TNF)-α* levels decreased over seven days in both groups, although the reduction of interleukin-6 was statistically significant. More reduction of IL-6 levels and ferritin was observed on the seventh day in group A, but the differences between the control group and intervention group were not statistically significant. The likelihood of higher *CRP* decreased during one week, but this decrement was not statistically significant. According to the trial outputs, a 7- day course of MTZ helped cure Covid-19 in cases with moderate disease. Such a helpful situation has been observed in a decrease of *IL-6* level and *ESR*, Ferritin, and *CRP *in comparison to the control group on the seventh day to the first day, which is the day of the beginning of cytokine storm. It should be mentioned that a window period has been seen during the clinical procedure of COVID-19 pneumonia between diagnosing and occurring multiple organ dysfunction syndromes (MODS) (~5 to 7 days). Following the mentioned window, many cases showed nearly 80% improvement while approximately 20% of the cases experienced severe pneumonia, and about 2% died (14).

Beforehand, some clinical trials suggested that MTZ is capable of decreasing the level of numerous cytokines, including interleukin IL-6 and tumor necrosis factor *(TNF)-α*, and level of *CRP* (15,16). The results of these studies were the same as this survey, although the decrease was not significant in all parameters may be due to the small sample size in current research. This dosage of MTZ (250 mg MTZ tablets orally every 6 hours) has been effective in the reduction of cytokine levels such as interleukin-1 and -6, and interleukin-8 in bacterial vaginosis during pregnancy (15). Modulation of cytokine production of *IL-6, IL-1β, IL-12*, *IL-8* as well as *TNF-α* on the humans’ periodontal ligament cells via MTZ was previously suggested (16). However, previous observations have shown that MTZ has an immunosuppressive effect, reduces neutrophil counts (17, 18, 19), and increases lymphocyte counts (18,19), but no significant differences in the present research were not observed in lymphocyte count in the group receiving MTZ.

The current study showed that MTZ inhibited ESR in patients with covid-19 but was unable to change oxygen saturation, hospital stay and mortality. As far as we know, no clinical studies have been performed on the clinical effects of MTZ in covid-19. We recommend that COVID-19 pneumonia cases received immuno-therapy when the disease was diagnosed for blocking probable consecutive cytokine storms and reduce mortality. It seems a higher sample size and longer follow-up in the future surveys demonstrate more clinical anti-inflammatory effect of MTZ in patients with COVID–19.

**Table 1:** Baseline and medical history of patients according to the study groups

***P*** **-value**	**Study group**		**Variable**
	**B** **(n = 24)**	**A** **(n = 20)**	
0.534	64.46 ± 18.91	61.26 ± 13.02	Age mean±SD
			Gender N (%)
0.911	14 (58.3)	12 (60)	male
	10 (41.7)	8 (40)	female
0.493	2 (8.3)	0 (0)	NIV N (%)
			PMD N (%)
0.757	7 (29.2)	5 (25)	Any
0.783	11 (45.8)	10 (50)	HTN
0.999	4 (16.7)	4 (20)	DM
0.472	4 (16.7)	6 (30)	IHD
0.999	1 (4.2)	1 (5)	CHF
0.646	2 (8.3)	3 (15)	CVA
0.999	1 (4.2)	0 (0)	ESRD
0.999	2 (8.3)	1 (5)	Obstructive airways disease
0.999	7 (29.2)	5 (25)	Another disease

**Table 2:** Antiviral Drug

***P*** **-value**	**Study group**		**Variable**
	**B**	**A**	
	N (%)	N (%)	Antiviral Drug
0.493	2 (8.3)	0 (0)	Any
0.911	14 (58.3)	12 (60)	Hydroxychlorhoqine
0.824	10 (41.7)	9 (45)	Kaletra
0.922	2 (8.3)	1 (5)	Ribavirine

**Table 3:** Description and comparison of Inflammatory factors at the begging and the end of study between the study groups

***P*** **-value** ^**^	**7** ^th^ ** day**	**1** ^st^ ** day**	**Group**	
0.836	38.25 ± 18.75	39.65 ± 29.22	A	ESR (mm/h)
0.003	47.67 ± 26.41	33.17 ± 19.50	B	
	0.021^‡^	0.385*		*P*-value
0.171	252.0 ± 211.4	333.1 ± 283.1	A	Ferritin (ng/mL)
0.188	313.2 ± 184.3	371.5 ± 278.5	B	
	0.351^‡^	0.524*		*P*-value
0.327	61.13 ± 13.95	70.02 ± 30.37	A	TNF-α (pg/mL)
0.427	80.45 ± 43.24	91.56 ± 71.40	B	
	0.260^‡^	0.320*		*P*-value
0.012	9.29 ± 18.04	49.35 ± 59.56	A	IL6 (pg/mL)
0.019	12.33 ± 26.68	34.37 ± 42.71	B	
	0.195^‡^	0.438*		*P*-value
0.492	699.3 ± 703.6	738.0 ± 1412.8	A	D.Dimer (ng/mL)
0.272	886.0 ± 1427.8^‡^	942.3 ± 3010.5	B	
	0.898	0.311*		*P*-value

^ *^ *P*-value on the basis of independent sample *t*-test. ^**^P-value on the basis of paired sample t-test. ^‡^ *P*-value on the basis of ANCOVA for comparison of

responses on 7^th^ day between the study groups adjusted for age, sex, , antiviral drugs and baseline value of responses.

**Table 4:** Description and comparison of study variables at the begging and the end of study between the study groups

***P*** **-value** ^**^	**7** ^th^ ** day**	**1** ^st^ ** day**	**Group**	**Variable**
0.009	93.50 ± 3.27	90.15 ± 4.23	A	Oxygen saturation (%)
0.001	93.54 ± 1.53	88.75 ± 6.76	B	
	0.818^‡^	0.546^*^		*P*-value
0.622	7.02 ± 2.00	7.26 ± 2.10	A	WBC(×103/µl)
0.783	6.57 ± 1.88	6.71 ± 2.51	B	
	0.612^‡^	0.305^*^		*P*-value
0.151	32.70 ± 17.27	26.70 ± 12.67	A	Lymphocyte(%)
0.022	28.62 ± 10.25	23.08 ± 11.09	B	
	0.422^‡^	0.389^*^		*P*-value
0.003	283.1 ± 87.73	211.9 ± 67.41	A	Platelet(×103/µl)
0.011	239.4 ± 79.24	206.1 ± 67.45	B	
	0.172^‡^	0.271^*^		*P*-value
0.948	11.87 ± 1.28	11.90 ± 2.17	A	PDW (%)
0.781	12.28 ± 2.50	12.17 ± 2.41	B	
	0.583^‡^	0.700^*^		*P*-value
0.821	9.38 ± 0.91	9.31 ± 1.18	A	MPV (fl)
0.340	9.42 ± 1.14	9.58 ± 1.13	B	
	0.999^‡^	0.459^*^		*P*-value
0.626	35.95 ± 28.54	39.60 ± 26.53	A	AST (U/L)
0.308	51.46 ± 98.64	155.0 ± 584.7	B	
	0.958^‡^	0.596*		*P*-value
0.485	31.80 ± 19.21	35.75 ± 32.87	A	ALT(U/L)
0.335	62.25 ±160.3	149.7 ± 594.7	B	
	0.573^‡^	0.398^*^		*P*-value
0.560	175.3 ± 57.10	179.7 ± 52.59	A	ALP (U/L)
0.374	203.3 ± 76.93	212.9 ± 86.11	B	
	0.994^‡^	0.191^*^		*P*-value
0.878	0.760 ± 0.298	0.745 ± 0.346	A	BIL (mg/dL)
0.730	0.704 ± 0.370	0.667 ± 0.324	B	
	0.069^‡^	0.443^*^		*P*-value
0.939	430.0 ± 161.4	427.1 ± 109.1	A	LDH (U/L)
0.994	418.3 ± 197.1	404.7 ± 164.2	B	
	0.725^‡^	0.128^*^		*P*-value

^ *^ *P*-value on the basis of independent sample *t*-test. ^**^P-value on the basis of paired sample *t*-test. ^‡^*P*-value on the basis of ANCOVA for comparison of

responses on 7^th^ day between the study groups adjusted for age, sex, antiviral drugs and baseline value of responses.

## Funding Support

As mentioned earlier, the Clinical ResearchDevelopment Center of LoghmanHakim Hospital, ShahidBeheshti University of Medical Sciences, has supported this trial. The study was funded by the Iranian Government.

## Conflicts of Interest

It is declared that there are not any conflicts of interest in the present research.
